# Vitamin D deficiency in adult fracture patients: prevalence and risk factors

**DOI:** 10.1007/s00068-015-0550-8

**Published:** 2015-07-21

**Authors:** E. A. Gorter, P. Krijnen, I. B. Schipper

**Affiliations:** Department of Surgery and Traumatology, Leiden University Medical Centre, K6-50, P.O. Box 9600, 2300 Leiden, The Netherlands

**Keywords:** Vitamin D, Vitamin D deficiency, Risk factors, Fracture, Fracture healing

## Abstract

**Purpose:**

Although vitamin D levels are not routinely monitored in outpatient fracture patients, identification of fracture patients with a deficient vitamin D status may be clinically relevant because of the potential role of vitamin D in fracture healing. This study aimed to determine the prevalence of and risk factors for vitamin D deficiency in non-operatively treated adult fracture patients.

**Patients and methods:**

Vitamin D levels were determined in a cross-sectional study of adult patients, who were treated non-operatively for a fracture of the upper or lower extremity in the outpatient clinic of a level 1 trauma center, during one calendar year. Potential risk factors for (severe) vitamin D deficiency were analyzed using multivariable logistic regression analysis.

**Results:**

A total of 208 men and 319 women with a mean age of 49.7 years (SD 19.9) were included. In this population, 71 % had a serum calcidiol <75 nmol/L, 40 % were vitamin D deficient (serum calcidiol <50 nmol/L) and 11 % were severely vitamin D deficient (serum calcidiol <25 nmol/L). Smoking and season (winter and spring) were independent risk factors for vitamin D deficiency. An increasing age, a non-Caucasian skin type, winter and smoking were identified as independent risk factors for severe vitamin D deficiency. The use of vitamin D, alcohol consumption and higher average daily sun exposure were independent protective factors against (severe) vitamin D deficiency.

**Conclusion:**

Given the potential role of vitamin D in fracture healing, clinicians treating adult fracture patients should be aware of the frequent presence of vitamin D deficiency during the winter, especially in smoking and non-Caucasian patients. Research on the effect of vitamin D deficiency or supplementation on fracture healing is needed, before suggesting routine monitoring or supplementation.

## Introduction

Vitamin D is acquired through nutritional uptake and by the cutaneous synthesis under the influence of UV radiation. Vitamin D status has been associated with cancer, immune deregulation, diabetes mellitus, cardiovascular health, muscle function and mental health [1]. Vitamin D is also essential for the development and maintenance of mineralized bone [2]. It plays a significant role in the complex cellular processes of fracture healing [3]. Although animal studies suggest that a deficiency may hamper fracture healing, human studies that address the clinical effects of vitamin D deficiency or supplementation on fracture healing are scarce and remain inconclusive [3].

The prevalence of vitamin D deficiency is considered a global health problem [4, 5]. In fracture patients, most studies focus on the elderly with hip fractures and predominantly osteoporotic fractures. These studies found a vitamin D deficiency (serum calcidiol <50 nmol/L) prevalence varying between 22 and 100 % [6–30]. Studies in non-hip or osteoporotic fracture patients found vitamin D prevalences of 13–50 % [31–36].

Currently, vitamin D status is not routinely monitored in outpatient fracture patients. Given the potential role that vitamin D has in fracture healing, it might be clinically relevant to identify fracture patients who are at risk for vitamin D deficiency. The aim of the present study was to determine the prevalence of vitamin D deficiency and identify risk factors for vitamin D deficiency in outpatient adult fracture patients that were treated non-operatively for a fracture of the upper or lower extremity.

## Patients and methods

### Study design and participants

Approval for this cross-sectional study was obtained from the institutional Medical Ethics Review Committee. All consecutive adult patients (≥18 years) with conservatively treated fractures of the upper or lower extremity, in the outpatient clinic of our level 1 trauma center between 1 September 2012 and 1 October 2013, were informed about the study. They were asked to participate within 1 week after the fracture had occurred and to provide written informed consent. After the patient’s consent was obtained, blood was taken, a questionnaire was filled out, and demographic and fracture characteristics were documented.

### Procedures

Blood was taken during the first outpatient control. The serum concentration calcidiol was measured using an electrochemiluminescence immunoassay (ECLIA) from Roche Diagnostics (Modular E170). The vitamin D serum concentration was defined as sufficient if the serum calcidiol level was ≥75 nmol/L (30 ng/ml); insufficient if the level was between 50 and 75 nmol/L; deficient if the level was <50 nmol/L (20 ng/ml) and severely deficient if the serum level was <25 nmol/L (10 ng/ml) [1, 37–40].

Included patients, unaware of their vitamin D status, completed a questionnaire on potentially relevant factors for vitamin D deficiency including medical history, medication and vitamin D usage prior to fracture. In the questionnaire, daily UV radiation exposure was defined as the average number of hours spent outdoors between 10.00 a.m. and 15.00 p.m. [1, 37, 40]. Also, the use of a solarium was questioned. Skin type was determined using the Fitzpatrick scale [41] (Type I: pale white skin, always burns, never tans; Type II: white skin, burns easily, tans minimally; Type III: white skin, burns moderately, tans uniformly; Type IV: light brown/moderate brown skin, burns minimally, always tans well; Type V: brown, rarely burns, tans profusely. Type VI: dark brown to black skin, never burns).

### Statistical analysis

Patient characteristics are presented as mean and standard deviation (SD) or as number (%). Patient groups were compared using the Student’s *t* test for continuous variables and the Chi-square test or Fisher’s exact test (when the expected count in any of the cells of the 2 × 2 contingency table was <5) for categorical data. Patient characteristics with a univariable association (*p* ≤ 0.10) with (severe) vitamin D deficiency were combined in a forward stepwise multivariable logistic regression analysis to identify independent risk factors for these conditions (*p*-to-enter <0.05 and *p*-to-remove >0.10). The predictive value of selected potential risk factors was expressed as the adjusted odds ratio (OR) with its corresponding 95 % confidence interval (CI). Statistical analysis was performed with SPSS software version 20 (SPSS, Inc., Chicago, IL, USA). *p* values <0.05 were considered to be statistically significant.

## Results

### Patient characteristics

A total of 902 patients, 412 men and 490 women (54 %) with a mean age of 47.8 years (SD 21.3), were eligible and approached for participation. Of these, 208 men and 319 women (61 %) with a mean age of 49.7 years (SD 19.9) agreed to participate. The most frequently encountered reasons for non-participation included reluctance to undergo a venipuncture and participation in another study.

**Fig. 1:**
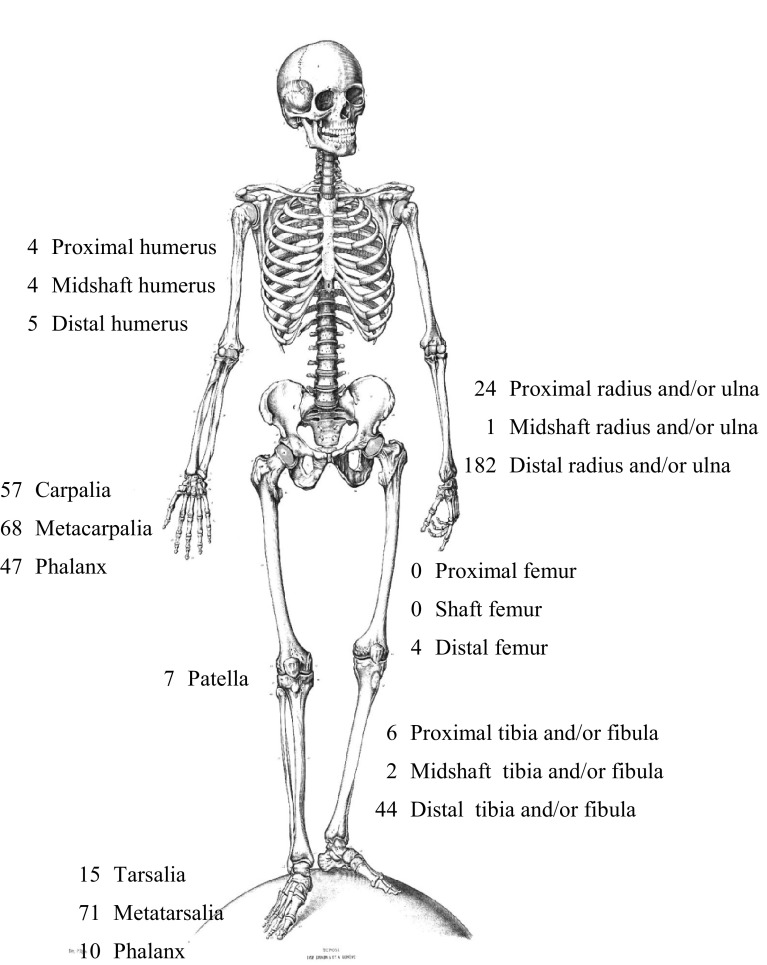
Number of fractures specified by location; 551 fractures in total

The vast majority of the 551 fractures in the 527 included patients were located in the upper extremity (71 %). The most frequent fractures were distal radius fractures (33 %; Fig. 1), followed by metatarsal (13 %) and metacarpal fractures (12 %). In the non-participating patient group, the fracture (*n* = 376) distribution was similar: 287 fractures were located in the upper extremity (76 %), 27 % in the distal radius, 21 % in the metacarpal bones and 10 % in the metatarsal bones.

Of the 527 patients, 101 (19 %) had no previous medical history, 216 (41 %) did not use any medication and 117 (22 %) used vitamin D supplements, in some cases in combination with calcium or as a component in a multivitamin (Table 1). Most patients, 482 (92 %), had a white skin type (I–III), 38 patients (7 %) had skin type IV and only 4 (1 %) had skin type V or VI. The average sun exposure between 10.00 a.m. and 15.00 p.m. was 1.9 h per day (SD 1.2).

**Table 1:** Patient characteristics and their univariable association with vitamin D deficiency (calcidiol <50 mol/L) and severe vitamin D deficiency (calcidiol <25 mol/L)

Characteristic	Total *n* = 527 (%)	Vitamin D deficiency			Severe vitamin D deficiency		
		Yes *n* = 210 (%)	No *n* = 317 (%)	*p*	Yes *n* = 58 (%)	No *n* = 469 (%)	*p*
Gender							
Female	319 (61)	118 (37)	201 (63)	**0.10**	33 (10)	286 (90)	0.55
Male	208 (39)	92 (44)	116 (56)		25 (12)	183 (88)	
Age in years, mean (SD)	49.7 (19.9)	51.5 (20.5)	48.6 (19.5)	**0.10**	53.8 (20.7)	49.2 (19.8)	**0.10**
BMI							
Normal/overweight; BMI <30	433 (82)	162 (37)	271 (63)	**0.05**	47 (11)	386 (89)	0.85
Obesity; BMI ≥30	71 (14)	37 (52)	34 (48)		9 (13)	62 (87)	
Unknown	23 (4)	11 (48)	12 (52)		2 (9)	21 (91)	
Skin type							
Caucasian (type I, II, III)	482 (92)	187 (39)	295 (61)	**0.04**	47 (10)	435 (90)	**0.003**
Non-Caucasian (type IV, V, VI)	42 (8)	23 (45)	19 (55)		11 (26)	31 (74)	
Medical history							
Medical history							
Yes	426 (81)	176 (41)	250 (59)	0.16	47 (11)	379 (89)	0.97
No	101 (19)	34 (34)	67 (66)		11(11)	90 (89)	
Previous fracture							
Yes	270 (51)	109 (40)	161 (60)	0.77	32 (12)	238 (88)	0.57
No	253 (49)	99 (39)	154 (61)		26 (10)	227 (90)	
Hypertension							
Yes	85 (16)	34 (40)	51 (60)	0.98	8 (9)	77 (91)	0.61
No	442 (84)	176 (40)	266 (60)		50 (11)	392 (89)	
Diabetes mellitus							
Yes	22 (4)	13 (59)	9 (41)	**0.06**	3 (14)	19 (86)	0.72
No	505 (96)	197 (39)	308 (61)		55 (11)	450 (89)	
Depression							
Yes	20 (4)	8 (40)	12 (60)	0.99	4 (20)	16 (80)	0.19
No	507 (96)	202 (40)	305 (60)		54 (11)	305 (98)	
Osteoporosis							
Yes	13 (2)	3 (23)	10 (77)	0.21	2 (15)	11 (85)	0.64
No	514 (98)	207 (40)	307 (60)		56 (11)	458 (89)	
Use of medication							
Vitamin D							
Yes	117 (22)	31 (27)	86 (73)	**0.001**	5 (4 %)	112 (96)	**0.008**
No	408 (78)	179 (44)	229 (56)		53 (13)	355 (87)	
Antihypertensive							
Yes	103 (20)	51 (50)	52 (50)	**0.03**	13 (13)	90 (87)	0.56
No	424 (80)	159 (38)	265 (62)		45 (11)	379 (89)	
NSAID							
Yes	34 (6)	20 (59)	14 (41)	**0.02**	7 (21)	27 (79)	**0.08**
No	493 (94)	190 (39)	303 (61)		51 (10)	442 (90)	
Antidepressive							
Yes	21 (4)	9 (43)	12 (57)	0.77	3 (14)	18 (86)	0.50
No	506 (96)	201 (40)	305 (60)		55 (11)	451 (89)	
Oral antidiabetics or insulin							
Yes	20 (4)	11 (55)	9 (45)	0.16	2 (10)	18 (90)	1.00
No	507 (96)	199 (39)	308 (61)		56 (11)	451 (89)	
Corticosteroids							
Yes	13 (3)	6 (46)	7 (56)	0.64	2 (15)	11 (85)	0.64
No	514 (97)	204 (40)	310 (60)		56 (11)	458 (89)	
Intoxication							
Smoking							
Yes	122 (24)	62 (51)	60 (49)	**0.005**	24 (20)	98 (80)	**0.001**
No	394 (76)	144 (37)	250 (63)		33 (8)	361 (92)	
Alcohol consumption None	200 (39)	102 (51)	98 (49)	**<0.001**	34 (17)	166 (83)	**0.002**
Alcohol consumption ≤2 U/day	274 (53)	96 (35)	178 (65)		21 (8)	253 (92)	
Alcohol consumption >2 U/day	45 (9)	10 (22)	35 (78)		2 (4)	43 (96)	
Sun exposure							
No. of hours/day, mean (SD)	1.9 (1.2)	1.7 (1.1)	2.1 (1.2)	**<0.001**	1.6 (1.0)	1.9 (1.2)	**0.02**
Use of solarium							
Yes	37 (7)	7 (19)	30 (81)	**0.007**	1 (3)	36 (97)	0.11
No	480 (93)	199 (41)	281 (59)				
Vacation in the prior 4 weeks							
Yes	60 (12)	11 (18)	49 (82)	**<0.001**	2 (3)	58 (97)	**0.04**
No	459 (88)	195 (42)	264 (58)				
Season of inclusion							
Summer	133 (25)	39 (29)	94 (71)	**<0.001**	10 (8)	123 (92)	**0.004**
Autumn	153 (29)	44 (29)	109 (71)		9 (6)	144 (94)	
Winter	122 (23)	64 (53)	58 (47)		22 (18)	100 (82)	
Spring	119 (23)	63 (53)	56 (47)		17 (14)	102 (86)	

Results are presented as number (% of non-missing cases) unless indicated otherwise

*SD* standard deviation, *BMI* body mass index, *NSAID* nonsteroidal antiinflammatory drug

*P* values in bold indicate a univariable association (*p* ≤ 0.10)

**Fig. 2:**
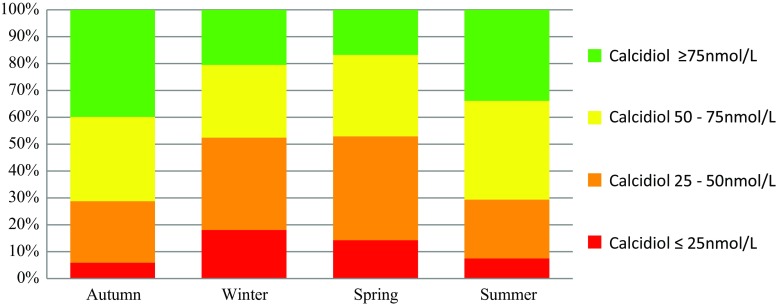
Vitamin D status in adult fracture patients by season: summer (June–August), autumn (September–November), winter (December–February) and spring (March–May)

The blood sample was taken at a median period of 7 days after fracture (range 0–85 days). The mean concentration serum calcidiol was 59·5 nmol/L (SD 29.4, range 8–175). A minority of 151 (29 %) patients had a sufficient calcidiol level, 166 patients (31 %) had insufficient levels (50–75 nmol/l) and 210 patients (40 %) had a vitamin D deficiency (calcidiol <50 nmol/L), of whom 58 patients (11 % of the total group) had a severe vitamin D deficiency. The highest prevalence of vitamin D deficiency was observed during the winter and spring (53 %; Fig. 2).

### Risk factors for vitamin D deficiency: calcidiol <50 nmol/L

Potential risk factors for vitamin D deficiency (univariable *p* ≤ 0.10) were male gender, older age, body mass index (BMI) ≥30, non-Caucasian skin type (skin type IV, V, VI), diabetes mellitus, use of antihypertensive medication or nonsteroidal antiinflammatory drugs (NSAIDs), smoking and season (winter and spring) (Table 1). Potentially protective factors against vitamin D deficiency were the use of vitamin D, alcohol consumption, more daily sun exposure and the use of a solarium or a holiday with high sun exposure within the previous month.

In the multivariable logistic regression model, independent and statistically significant risk factors for vitamin D deficiency were smoking (OR 2.02) and season (winter OR 2.44 and spring OR 3.07) (Table 2). Independent protective factors against vitamin D deficiency were the use of vitamin D (OR 0.46), alcohol consumption (OR 0.47 and 0.26 respectively for ≤2 and >2 units per day), more daily sun exposure (OR 0.77 per additional hour) and a recent holiday with a high sun exposure (OR 0.43).

**Table 2:** Multivariable logistic regression analysis including potential risk factors (univariable *p* ≤ 0.10) for vitamin D deficiency (calcidiol <50 mol/L) and severe vitamin D deficiency (calcidiol <25 mol/L)

Characteristic	Vitamin D deficiency Adjusted OR (95 % CI)	Severe vitamin D deficiency Adjusted OR (95 % CI)
Gender		
Women	Not included	–
Men		
Age		
Years	1.01 (1.00–1.02)	1.02 (1.01–1.04)
BMI		
Normal/overweight; BMI <30	Not included	–
Obese; BMI ≥30		
Unknown		
Skin type		
Caucasian (type I, II, III)	Not included	Reference
Non-Caucasian (type IV, V, VI)		4.07 (1.63–10.2)
Medical history		
Diabetes mellitus		
Yes	Not included	–
No		
Use of medication		
Vitamin D		
Yes	0.46 (0.28–0.77)	0.28 (0.10–0.79)
No	Reference	Reference
Antihypertensive		
Yes	Not included	–
No		
NSAID		
Yes	Not included	Not included
No		
Intoxication		
Smoking		
Yes	2.02 (1.25–3.25)	2.79 (1.44–5.42)
No	Reference	Reference
Alcohol consumption, none	Reference	Reference
Alcohol consumption ≤2 U/day	0.47 (0.31–0.71)	0.42 (0.22–0.79)
Alcohol consumption >2 U/day	0.26 (0.11–0.62)	0.24 (0.06–1.13)
Sun exposure		
Number of hours/day	0.77 (0.65–0.92)	0.72 (0.54–0.97)
Use of solarium		
Yes	0.40 (0.15–1.00)	–
No	Reference	
Vacation in the prior 4 weeks		
Yes	0.43 (0.21–0.89)	Not included
No	Reference	
Season of inclusion		
Summer	Reference	Reference
Autumn	1.09 (0.62–1.93)	0.81 (0.29–2.23)
Winter	2.44 (1.36–4.38)	2.61 (1.01–6.17)
Spring	3.07 (1.70–5.55)	2.23 (0.90–5.49)

*BMI* body mass index, *NSAID* nonsteroidal antiinflammatory drug, *OR* odds ratio, *CI* confidence interval, *X* Not included in the analysis (univariable *p* > 0.10), *Not included* not included in the logistic regression model after forward stepwise selection

### Risk factors for severe vitamin D deficiency: calcidiol <25 nmol/L

Potential risk factors for severe vitamin D deficiency (univariable *p* ≤ 0.10) were older age, non-Caucasian skin type, use of NSAIDs, smoking and season (winter and spring) (Table 1). Potentially protective factors against severe vitamin D deficiency were use of vitamin D, alcohol consumption, more daily sun exposure and a recent holiday with high sun exposure.

In the multivariable logistic regression model, independent risk factors for severe vitamin D deficiency were older age (OR 1.02 per 1-year increase), non-Caucasian skin type (OR 4.07), smoking (OR 2.79) and winter (OR 2.61) (Table 2). Independent protective factors against severe vitamin D deficiency were the use of vitamin D (OR 0.28), limited alcohol consumption (≤2 alcohol units per day OR 0.42) and more daily sun exposure (OR 0.72 per additional hour).

## Discussion

Our results shows that on average in one calendar year, 71 % of the outpatient adult fracture population had a suboptimal vitamin D status (calcidiol <75 nmol/L), 40 % was vitamin D deficient and 11 % was severely vitamin D deficient. Smoking and season (winter and spring) were independent risk factors for a vitamin D deficiency, whereas smoking, winter, age and a non-Caucasian skin type were identified as independent risk factors for severe vitamin D deficiency.

We defined vitamin D deficiency as a serum calcidiol level <50 nmol/L, and ≥75 nmol/L was considered to be optimal/sufficient. These commonly used cutoff values are based on studies evaluating the effect of calcidiol concentration on calcium absorption, parathyroid hormone synthesis suppression, maintenance of bone mineral density and fall/fracture prevention and other non-skeletal actions of vitamin D [1, 37–39, 42]. However, due to the inconsistent evidence regarding these effects, there is no consensus in literature on these definitions [1, 4, 37, 43–46]. It has also been suggested that a serum calcidiol >50 nmol/L could be sufficient.

Compared to other studies in non-hip or osteoporotic fracture patients, we found seeming differences in prevalence (Table 3). Briggs et al. [33] found a vitamin D deficiency in 14/28 fracture patients in London between April and October. In our region (latitude 52°N) we found a 33 % deficiency prevalence during these months. Bee et al. [34] measured a serum calcidiol <50 nmol/L in 28 % of their operated fracture population, 32/103 (31 %) during the winter (January, February and March) and 24/98 (26 %) during summer (July, August and September). In these periods, 50 and 24 % of our patients were deficient, respectively. Wright et al. [31] found in 18/37 (49 %) male patients with a distal forearm fracture living in Northern Ireland a vitamin D deficiency. Of the 49 male patients with a distal forearm fracture in our study, 20 (41 %) were vitamin D deficient. Smith et al. [35] found a deficiency only in 10/75 (13 %) patients with an ankle fracture, as compared to 53 % of the patients with an ankle fracture in our study. Four of our seven patients with patellar fractures were vitamin D deficient, where Reinhardt et al. [36] found a prevalence of 33 %. Bogunovic et al. [32] also found a 40 % vitamin D deficiency in their operated trauma patients, and 94/121 patients had a lower extremity fracture. These seeming differences in prevalence may have resulted from seasonal differences and differences in geographical distribution or latitude [1, 4], but may also be caused by differences in other characteristics of the study populations and by statistical imprecision. Nonetheless, clinicians should be aware that patients living north of 35 degrees latitude produce little or no vitamin D from November to February due to the scarce sunlight [1], and uptake from food is generally insufficient to retain adequate serum concentrations of vitamin D [37]. Consequently, active supplementation is the only way for vitamin D-deficient patients to complement their deficiency during the winter months.

**Table 3:** Prevalence of vitamin D deficiency (calcidiol <50 mol/L) in adult fracture patients

References	Year	Location—latitude	Fracture population	*n*	Mean age	Vitamin D deficiency (%)
Bee et al. [34]	2013	USA; 42–44°N	Operated traumatic fractures in the upper and lower extremities	201	56	28
Briggs et al. [33]	2013	UK; 51°N	Operated and non-operated long bone fractures	33	53	50
Bogunovic et al. [32]	2010	USA; 41°N	Operated traumatic fractures in the upper and lower extremities	121	63	40
Bogunovic et al. [32]	2010	USA; 41°N	Operated distal radial or ulnar fracture	43	66	16
Reinhardt et al. [36]	2012	USA; 41°N	Operated patella fractures	30	58	33
Smith et al. [35]	2013	USA; 42°N	Ankle fractures	75	52	13
Wright et al. [31]	2007	UK; 55°N	Forearm fracture in males	37	54	49

*UK* United Kingdom, *USA* United States of America

Given the role of vitamin D in the maintenance of bone health, it could be hypothesized that vitamin D deficiency might be more prevalent in a fracture population than in the general population. However, the prevalence of 40 % in our fracture population was relatively low compared to estimates in the general Dutch population (43–71 %) [47–49]. Well-known causes of vitamin D deficiency include reduced skin synthesis (skin type, sun exposure, aging, season and latitude), decreased bio-availability (malabsorption, obesity), decreased synthesis (liver failure), increased catabolism and increased urinary loss [1]. Of these causes, the predictive value of skin type, sun exposure, aging, season and obesity was analyzed and confirmed in our study, although statistical significance could not demonstrate all parameters. This may be due to the small patient numbers for some subgroups such as obese patients and patients with a non-Caucasian skin type.

In concordance with the results of earlier studies [35, 50, 51], vitamin D deficiency was more prevalent in smokers in our study. Smoking has been shown to delay fracture healing in animal and human studies and nicotine is thought to inhibit the vascularization of bone and diminish osteoblast function [52]. On the other hand, vitamin D has been shown to modulate the synthesis of vascular growth factors [53] and functioning of osteoblasts [54, 55]. The combination of the direct (vascularization) and indirect effects (vitamin D deficiency) of smoking might substantially increase the risk for impaired bone healing, although this hypothesis is still to be confirmed in further research.

A striking finding was that the use of alcohol was associated with a reduced risk of vitamin D deficiency in our study group. This association has been found in other studies [50], whereas other studies did not find any association [49, 56–59], or a negative association [60–62]. As yet, the mechanism by which alcohol may affect the serum concentration calcidiol remains rather unknown. Some studies indicate that alcohol influences the serum concentration vitamin D indirectly through its effect on the expression of parathyroid hormone [59, 63, 64]. On the other hand, results from an animal study showed that alcohol results in CYP_24_A1 induction, an enzyme that breaks down calcidiol [65].

A limitation of this study is that only the serum concentration calcidiol was measured and not serum 1,25-dihydroxycholecalciferol or vitamin D binding protein. Calcidiol is considered to be the best indicator to monitor the vitamin D status, as 1,25-dihydroxycholecalciferol does not reflect vitamin D reserves or vitamin D status [37]. However, as the most active form of vitamin D, 1,25-dihydroxycholecalciferol might reflect vitamin D activity during the initial phase of fracture healing better. A low vitamin D binding protein is found to compensate for a low serum concentration of calcidiol (deficiency) resulting in similar “net” concentrations of estimated bio-availability calcidiol [66]. Another study limitation was that the time until blood sampling ranged up to 85 days, although half of the samples were obtained within 7 days and 80 % within 11 days after the fracture. The majority of the cases with delayed blood sampling occurred in patients who were referred from other hospitals and some other cases were due to a delayed inclusion in the study. These delayed blood samples may have resulted in a less accurate determination of the vitamin D status at the time of fracture, taking into account the circulating half-life of calcidiol (2–3 weeks) and its metabolization during the process of fracture healing. Vitamin D status should ideally be determined on the day of fracture. Another issue is that the percentage of women and mean age in the study group were somewhat higher compared to the non-participating patients, which may affect the generalizability of the study results. These differences were most likely due to the fact that patients above the age of 50 years were routinely offered a screening for osteoporosis, and the proportion of women in this age category was higher.

In conclusion, we found 71 % of our adult fracture patients to have suboptimal levels of vitamin D, including 40 % with vitamin D deficiency (calcidiol <50 nmol/L). Given the potential of vitamin D in fracture healing, clinicians treating adult fracture patients should be aware of the frequent presence of vitamin D deficiency during the winter, especially in smoking and non-Caucasian patients. Research on the effect of vitamin D deficiency and supplementation on fracture healing is needed, before suggesting routine monitoring or supplementation in all adult fracture patients or in selected groups.

## References

[1] Holick MF (2007). Vitamin D deficiency. N Engl J Med.

[2] Dusso AS, Brown AJ, Slatopolsky E (2005). Vitamin D. Am J Physiol Renal Physiol.

[3] Gorter EA, Hamdy NA, Appelman-Dijkstra NM, Schipper IB (2014). The role of vitamin D in human fracture healing: a systematic review of the literature. Bone.

[4] Mithal A, Wahl DA, Bonjour JP, Burckhardt P, Dawson-Hughes B, Eisman JA (2009). Global vitamin D status and determinants of hypovitaminosis D. Osteoporos Int.

[5] Wahl DA, Cooper C, Ebeling PR, Eggersdorfer M, Hilger J, Hoffmann K (2012). A global representation of vitamin D status in healthy populations. Arch Osteoporos.

[6] Dhanwal DK, Sahoo S, Gautam VK, Saha R (2013). Hip fracture patients in India have vitamin D deficiency and secondary hyperparathyroidism. Osteoporos Int.

[7] Fisher A, Srikusalanukul W, Davis M, Smith P (2010). Hip fracture type: important role of parathyroid hormone (PTH) response to hypovitaminosis D. Bone.

[8] Moniz C, Dew T, Dixon T (2005). Prevalence of vitamin D inadequacy in osteoporotic hip fracture patients in London. Curr Med Res Opin.

[9] Nurmi I, Kaukonen JP, Luthje P, Naboulsi H, Tanninen S, Kataja M (2005). Half of the patients with an acute hip fracture suffer from hypovitaminosis D: a prospective study in southeastern Finland. Osteoporos Int.

[10] Breuil V, Roux CH, Testa J, Albert C, Chassang M, Brocq O (2008). Outcome of osteoporotic pelvic fractures: an underestimated severity. Survey of 60 cases. Joint Bone Spine.

[11] Khadgawat R, Brar KS, Gahlo M, Yadav CS, Malhotra R, Guptat N (2010). High prevalence of vitamin D deficiency in Asian–Indian patients with fragility hip fracture: a pilot study. J Assoc Physicians India.

[12] Nuti R, Martini G, Valenti R, Gambera D, Gennari L, Salvadori S (2004). Vitamin D status and bone turnover in women with acute hip fracture. Clin Orthop Relat Res.

[13] Sakuma M, Endo N, Oinuma T, Hayami T, Endo E, Yazawa T (2006). Vitamin D and intact PTH status in patients with hip fracture. Osteoporos Int.

[14] Sakuma M, Endo N, Hagino H, Harada A, Matsui Y, Nakano T (2011). Serum 25-hydroxyvitamin D status in hip and spine-fracture patients in Japan. J Orthop Sci.

[15] van den Bergh J, van Geel T, Geusens P (2010). Should the vitamin D level be determined for all fracture patients?. Ned Tijdschr Geneeskd.

[16] Bakhtiyarova S, Lesnyak O, Kyznesova N, Blankenstein MA, Lips P (2006). Vitamin D status among patients with hip fracture and elderly control subjects in Yekaterinburg. Russ Osteoporos Int.

[17] Gallacher SJ, McQuillian C, Harkness M, Finlay F, Gallagher AP, Dixon T (2005). Prevalence of vitamin D inadequacy in Scottish adults with non-vertebral fragility fractures. Curr Med Res Opin.

[18] Maier S, Sidelnikov E, Dawson-Hughes B, Egli A, Theiler R, Platz A (2013). Before and after hip fracture, vitamin D deficiency may not be treated sufficiently. Osteoporos Int.

[19] Bischoff-Ferrari HA, Can U, Staehelin HB, Platz A, Henschkowski J, Michel BA (2008). Severe vitamin D deficiency in Swiss hip fracture patients. Bone.

[20] Tanriover MD, Oz SG, Tanriover A, Kilicarslan A, Turkmen E, Guven GS (2010). Hip fractures in a developing country: osteoporosis frequency, predisposing factors and treatment costs. Arch Gerontol Geriatr.

[21] Dixon T, Mitchell P, Beringer T, Gallacher S, Moniz C, Patel S (2006). An overview of the prevalence of 25-hydroxy-vitamin D inadequacy amongst elderly patients with or without fragility fracture in the United Kingdom. Curr Med Res Opin.

[22] Becker C, Crow S, Toman J, Lipton C, McMahon DJ, Macaulay W (2006). Characteristics of elderly patients admitted to an urban tertiary care hospital with osteoporotic fractures: correlations with risk factors, fracture type, gender and ethnicity. Osteoporos Int.

[23] LeBoff MS, Kohlmeier L, Hurwitz S, Franklin J, Wright J, Glowacki J (1999). Occult vitamin D deficiency in postmenopausal US women with acute hip fracture. JAMA.

[24] LeBoff MS, Hawkes WG, Glowacki J, Yu-Yahiro J, Hurwitz S, Magaziner J (2008). Vitamin D-deficiency and post-fracture changes in lower extremity function and falls in women with hip fractures. Osteoporos Int.

[25] Simonelli C, Weiss TW, Morancey J, Swanson L, Chen YT (2005). Prevalence of vitamin D inadequacy in a minimal trauma fracture population. Curr Med Res Opin.

[26] Beringer T, Heyburn G, Finch M, McNally C, McQuilken M, Duncan M (2006). Prevalence of vitamin D inadequacy in Belfast following fragility fracture. Curr Med Res Opin.

[27] Shab-Bidar S, Bours SP, Geusens PP, van der Velde RY, Janssen MJ, van den Bergh JP (2013). Suboptimal effect of different vitamin D3 supplementations and doses adapted to baseline serum 25(OH) D on achieved 25(OH) D levels in patients with a recent fracture: a prospective observational study. Eur J Endocrinol.

[28] Kolb JP, Schilling AF, Bischoff J, de Novo OA, Spiro A, Hoffmann M (2013). Calcium homeostasis influences radiological fracture healing in postmenopausal women. Arch Orthop Trauma Surg.

[29] Oyen J, Apalset EM, Gjesdal CG, Brudvik C, Lie SA, Hove LM (2011). Vitamin D inadequacy is associated with low-energy distal radius fractures: a case–control study. Bone.

[30] Jang WY, Chung MS, Baek GH, Song CH, Cho HE, Gong HS (2012). Vitamin D levels in post-menopausal Korean women with a distal radius fracture. Injury.

[31] Wright S, Beringer T, Taggart H, Keegan D, Kelly J, Whithead E (2007). A study of male patients with forearm fracture in Northern Ireland. Clin Rheumatol.

[32] Bogunovic L, Kim AD, Beamer BS, Nguyen J, Lane JM (2010). Hypovitaminosis D in patients scheduled to undergo orthopaedic surgery: a single-center analysis. J Bone Joint Surg Am.

[33] Briggs AD, Kuan V, Greiller CL, Maclaughlin BD, Ramachandran M, Harris T (2013). Longitudinal study of vitamin D metabolites after long bone fracture. J Bone Miner Res.

[34] Bee CR, Sheerin DV, Wuest TK, Fitzpatrick DC (2013). Serum vitamin D levels in orthopaedic trauma patients living in the northwestern United States. J Orthop Trauma.

[35] Smith JT, Halim K, Palms DA, Okike K, Bluman EM, Chiodo CP (2013). Prevalence of vitamin d deficiency in patients with foot and ankle injuries. Foot Ankle Int.

[36] Reinhardt KR, Lazaro LE, Umunna BP, Cross MB, Helfet DL, Lane JM (2013). Plasma 25-hydroxyvitamin D levels in operative patella fractures. HSS J.

[37] Holick MF, Binkley NC, Bischoff-Ferrari HA, Gordon CM, Hanley DA, Heaney RP (2011). Evaluation, treatment, and prevention of vitamin D deficiency: an Endocrine Society clinical practice guideline. J Clin Endocrinol Metab.

[38] Bischoff-Ferrari H (2009). Vitamin D: what is an adequate vitamin D level and how much supplementation is necessary?. Best Pract Res Clin Rheumatol.

[39] Wimalawansa SJ (2012). Vitamin D in the new millennium. Curr Osteoporos Rep.

[40] Holick MF (2006). High prevalence of vitamin D inadequacy and implications for health. Mayo Clin Proc.

[41] Pathak MA, Fitzpatrick TB, Fitzpatrick TB, Eisen AZ, Wolff K (1993). Preventive treatment of sunburn, dermatoheliosis, and skin cancer with sun protective agents. Dermatology in general medicine.

[42] Bischoff-Ferrari HA, Giovannucci E, Willett WC, Dietrich T, Dawson-Hughes B (2006). Estimation of optimal serum concentrations of 25-hydroxyvitamin D for multiple health outcomes. Am J Clin Nutr.

[43] Brouwer-Brolsma EM, Bischoff-Ferrari HA, Bouillon R, Feskens EJ, Gallagher CJ, Hypponen E (2013). Vitamin D: do we get enough? A discussion between vitamin D experts in order to make a step towards the harmonisation of dietary reference intakes for vitamin D across Europe. Osteoporos Int.

[44] Rosen CJ, Abrams SA, Aloia JF, Brannon PM, Clinton SK, Durazo-Arvizu RA (2012). IOM committee members respond to Endocrine Society vitamin D guideline. J Clin Endocrinol Metab.

[45] Weggemans RM, Schaafsma G, Kromhout D (2009). Towards an adequate intake of vitamin D. An advisory report of the Health Council of the Netherlands. Eur J Clin Nutr.

[46] Ross AC (2011). The 2011 report on dietary reference intakes for calcium and vitamin D. Public Health Nutr.

[47] Deckers MM, de Jongh RT, Lips PT, Penninx BW, Milaneschi Y, Smit JH (2013). Prevalence of vitamin D deficiency and consequences for PTH reference values. Clin Chim Acta.

[48] van Dam RM, Snijder MB, Dekker JM, Stehouwer CD, Bouter LM, Heine RJ (2007). Potentially modifiable determinants of vitamin D status in an older population in the Netherlands: the Hoorn study. Am J Clin Nutr.

[49] Janssen HC, Emmelot-Vonk MH, Verhaar HJ, van der Schouw YT (2013). Determinants of vitamin D status in healthy men and women aged 40–80 years. Maturitas.

[50] Larose TL, Chen Y, Camargo CA, Langhammer A, Romundstad P, Mai XM (2013). Factors associated with vitamin D deficiency in a Norwegian population: the HUNT study. J Epidemiol Community Health.

[51] Rapuri PB, Gallagher JC, Balhorn KE, Ryschon KL (2000). Smoking and bone metabolism in elderly women. Bone.

[52] Brinker MR. Nonunions: evaluation and treatment. In: Browner BD, Levine AM, Jupiter JB, Trafton PG, editors. Skeletal trauma: basic science, management, and reconstruction. Saunders; 2002. p. 507–604.

[53] Gurlek A, Pittelkow, Kumar R (2002). Modulation of growth factor/cytokine synthesis and signaling by 1α, 25-dihydroxyvitamin D3: implications in cell growth and differentiation. Endocr Rev.

[54] van Driel M, Koedam M, Buurman CJ, Roelse M, Weyts F, Chiba H (2006). Evidence that both 1α, 25-dihydroxyvitamin D3 and 24-hydroxylated D3 enhance human osteoblast differentiation and mineralization. J Cell Biochem.

[55] van Leeuwen JP, van Driel M, van den Bemd GJ, Pols HA (2001). Vitamin D control of osteoblast function and bone extracellular matrix mineralization. Crit Rev Eukaryot Gene Expr.

[56] Bikle DD, Genant HK, Cann C, Recker RR, Halloran BP, Strewler GJ (1985). Bone disease in alcohol abuse. Ann Intern Med.

[57] Kuhn T, Kaaks R, Teucher B, Hirche F, Dierkes J, Weikert C (2014). Dietary, lifestyle, and genetic determinants of vitamin D status: a cross-sectional analysis from the European Prospective Investigation into Cancer and Nutrition (EPIC)-Germany study. Eur J Nutr.

[58] Hirani V, Cumming RG, Blyth FM, Naganathan V, Le Couteur DG, Handelsman DJ (2013). Vitamin D status among older community dwelling men living in a sunny country and associations with lifestyle factors: the Concord Health and Ageing in Men Project, Sydney, Australia. J Nutr Health Aging.

[59] Rapuri PB, Gallagher JC, Balhorn KE, Ryschon KL (2000). Alcohol intake and bone metabolism in elderly women. Am J Clin Nutr.

[60] Sobral-Oliveira MB, Faintuch J, Guarita DR, Oliveira CP, Carrilho FJ (2011). Nutritional profile of asymptomatic alcoholic patients. Arq Gastroenterol.

[61] Naude CE, Carey PD, Laubscher R, Fein G, Senekal M (2012). Vitamin D and calcium status in South African adolescents with alcohol use disorders. Nutrients.

[62] Santori C, Ceccanti M, Diacinti D, Attilia ML, Toppo L, D’Erasmo E (2008). Skeletal turnover, bone mineral density, and fractures in male chronic abusers of alcohol. J Endocrinol Invest.

[63] Sampson HW (1997). Alcohol, osteoporosis, and bone regulating hormones. Alcohol Clin Exp Res.

[64] McCarty MF, Thomas CA (2003). PTH excess may promote weight gain by impeding catecholamine-induced lipolysis-implications for the impact of calcium, vitamin D, and alcohol on body weight. Med Hypotheses.

[65] Shankar K, Liu X, Singhal R, Chen JR, Nagarajan S, Badger TM (2008). Chronic ethanol consumption leads to disruption of vitamin D3 homeostasis associated with induction of renal 1,25 dihydroxyvitamin D3-24-hydroxylase (CYP_24_A1). Endocrinology.

[66] Powe CE, Evans MK, Wenger J, Zonderman AB, Berg AH, Nalls M (2013). Vitamin D-binding protein and vitamin D status of black Americans and white Americans. N Engl J Med.

